# Cholesterol reducer and thrombolytic therapy in acute ischemic stroke patients

**DOI:** 10.1186/s12944-020-01270-2

**Published:** 2020-05-06

**Authors:** Nicolas Poupore, Dan Strat, Tristan Mackey, Katherine Brown, Ashley Snell, Thomas I. Nathaniel

**Affiliations:** University of South Carolina School of Medicine Greenville, 607 Grove Rd, Greenville, SC 29605 USA

**Keywords:** Cholesterol reducer, NIHSS score, Neurological deficits, Thrombolytic therapy, Ischemic stroke

## Abstract

**Background:**

Specific clinical risk factors may contribute to improving or worsening neurological functions in acute ischemic stroke (AIS) patients pre-treated with a combined cholesterol reducer and recombinant tissue plasminogen activator (rtPA) therapy. In this study, clinical risk factors associated with good or poor presenting neurological symptoms in ischemic stroke patients with prior cholesterol reducer use, specifically a statin and rtPA therapy was investigated.

**Methods:**

Retrospective data for baseline clinical and demographic data for patients with AIS taking cholesterol reducers prior to rtPA treatment from January 2010 to June 2016 in a regional stroke center was analyzed. Improving (NIHSS score ≤ 7) or worsening (NIHSS score > 7) of neurologic functions were the determined measures of treatment outcome. Multivariate logistic regression models identified demographic and clinical factors associated with worsening or improving neurologic functions.

**Results:**

Adjusted multivariate analysis showed that in an AIS population with a combined rtPA and cholesterol reducer medication history, increasing age (OR = 1.032, 95% CI, 1.015–1.048, *P* < 0.001) and atrial fibrillation (OR = 1.859, 95% CI, 1.098–3.149, *P* = 0.021) demonstrated a likely association with worsening neurologic functions, while direct admission (OR = 0.411, 95% CI, 0.246–0.686, *P* = 0.001) and being Caucasian (OR = 0.496, 95% CI, 0.297–0.827, *P* = 0.007) showed an association with improving or progressing neurologic functions.

**Conclusion:**

A prior cholesterol reducer, namely a statin, plus rtPA combination may be associated with worsening neurological function for elderly AIS patients with atrial fibrillation, while Caucasians directly admitted to a neurology unit are more likely to show an association with progress or improvements in neurologic functions. While combining statin with rtPA treatment may facilitate worsening neurologic functions in elderly AIS patients with atrial fibrillation, they should not be denied of this therapy. The decision to combine statin and rtPA for AIS patients with atrial fibrillation can be done after clinical stabilization following appropriate clinical management.

## Introduction

The function of cholesterol reducers, such as statins in the primary and secondary prevention of stroke in patients at risk of cerebrovascular events is well established [1–3]. Findings reveal that statins reduce the risk of first and recurrent ischemic strokes [4, 5] and may also improve treatment outcomes through pleiotropic non-statin-associated effects [6]. Several studies [7–12] report the relationship between statin use and stroke, including related treatment outcomes. While some studies [13–15] report contradictory evidence that cholesterol reducers, including statins, do not reduce initial stroke severity, other studies [16–18] report favorable outcomes associated with statin use and stroke. Findings from the aforementioned studies suggest that specific clinical risk factors may combine with the statins to contribute to either the reported favorable or unfavorable outcomes.

Patients with a National Institute of Health Stroke Scale (NIHSS) score of equal to or less than 7 at the time of admission demonstrate a higher likelihood of making positive neurological progress after an AIS, while NIHSS scores greater than 7 on admission demonstrated a higher probability of worsening neurological function in hemispheric strokes [19, 20]. Therefore, the baseline NIHSS, stratified by NIHSS scores of ≤7 and > 7 can be used to assess improvement or non-improvement following thrombolytic therapy for AIS [21, 22]. *NIHSS* scores have shown *to be predictive* for any vessel occlusions in the anterior circulation, and c*ut-off values* of *NIHSS* scores *> 7* from three *to* 6 h of stroke onset provided a *predictive value of* 84.4% for worsening neurologic functions [22]. In another study [21], major neurological improvements were observed with a NIHSS score of below eight within 24 h of stroke onset, while a score of ≤6 forecasts a favorable neurological recovery [23]. Although low and high NIHSS score cut-off points are effective predictors for mild or severe neurological outcomes, the NIHSS score is heavily weighted towards hemispheric strokes [24]. Therefore, the current study is focused on clinical risk factors associated with positive or poor presenting neurological symptoms in hemispheric ischemic stroke patients with prior cholesterol reducer use. Specifically, the focus is on a prior statin regimen and recombinant tissue plasminogen activator (rtPA) therapy. Recombinant tissue plasminogen activator is an approved Food and Drug Administration (FDA) thrombolytic agent for the treatment of AIS [25–27], resulting in improved functional outcome in patients treated soon after an AIS [28–36].

Eligibility for rtPA within a population of patients with AIS ranges from 6 to 8% [12, 13]. This suggests that specific clinical risk factors may contribute to stroke severity and affect treatment outcome following thrombolytic therapy with or without cholesterol reduction therapy [37–40]. The proportion of ischemic stroke patients with prior cholesterol reduction therapy and treated with rtPA at the time of admission may be different from those lacking prior cholesterol reduction therapy and treated with rtPA. This is possible if more clinical risk factors are associated with improving or worsening neurologic functions following rtPA in patients pretreated with a cholesterol reducer compared to those without a cholesterol reducer. The current study tested the hypothesis that specific clinical risk factors may contribute to improving or worsening neurologic functions in AIS patients with previous cholesterol reducer therapy who were treated with rtPA. Understanding and identifying the specific clinical risk factors associated with worsening neurologic functions associated with a combined rtPA and cholesterol reduction therapy will provide information about the measurable and qualitative risks of rtPA and cholesterol reducer use in ischemic stroke patients. In turn, this could help identify future potential research areas to investigate in order to increase eligibility for rtPA and improve ischemic stroke treatment outcomes.

## Methods

### Study population

This IRB approved (approval number: 00052571) retrospective study analyzed patients with AIS admitted to the health system of Prisma Health-Upstate in Greenville, South Carolina, USA between January 2010 and June 2016. The analysis included patients that presented within 24 h of symptom onset with an AIS based on brain magnetic resonance image (MRI) or computer tomography (CT) findings demonstrating early signs of ischemia (loss of gray/white matter distinction, hypodensity, or sulcal swelling) or middle cerebral artery (MCA) hyperdensity. Cholesterol reducer use, specifically a statin in this study, includes an active use at the time of admission, while patients that were not receiving a cholesterol reducer at the time of admission were considered as non-cholesterol reducer users. The stroke registry provided the data on demographics, clinical characteristics, and laboratory values and our previous studies have described the stroke registry [34, 35, 41]. Clinical characteristics of the patients included: atrial fibrillation/atrial flutter, coronary artery disease (CAD), carotid stenosis, depression, diabetes, drug or alcohol abuse, dyslipidemia, family history of stroke, congestive heart failure (CHF), hormone replacement therapy (HRT), hypertension, migraine, obesity, prior stroke, prior trans ischemic attack (TIA), prosthetic heart valve, peripheral vascular disease (PVD), chronic renal disease, sickle cell, sleep apnea, and history of smoking. This study also analyzed patients’ ambulatory data. The scores ranged from 0 to 3 in this fashion: undocumented (0), patients not able to ambulate (1), able to ambulate with assistance (2), and able to ambulate independently (3). The validity of the scoring has been described in previous studies [42–45]. Each patients’ ambulatory status was tracked and collected on admission, during admission, and after discharge. Ambulation was analyzed at discharge and compared with admission to discern any patient improvement in ambulation. In addition, data were collected on patient demographic variables, which included race, gender, ethnicity, BMI, medication history, and stroke severity (NIHSS) score.

### Statistical analysis

All statistical analyses were determined using the Statistical Package for Social Sciences v 26.0 for Windows (SPSS, Chicago, IL). The differences between the 2 groups stratified according to cholesterol reducer use prior to the stroke and by stroke severity, with NIHSS scores of > 7 representing worsening neurologic functions and NIHSS scores ≤7 represent neurologic improvement, was determined. Univariate statistical analysis was performed to determine factors associated with neurologic improvement or worsening stratified by rtPA and prior cholesterol reducer use. In the univariate analysis, the Pearson χ2 test analyzed discrete variables while the Student’s t test analyzed data for all continuous variables. To identify independent predictors of neurologic improvement or worsening, multivariate analyses were then performed to establish predictors (demographic and clinical variables) from the variables in the univariate analysis that had a probability value *P* < 0.2 [46]. Because of the nonrandomized design of the study, a post hoc adjusted analysis using logistic regression was performed to determine the most influential variables using the backward selection method. This approach allowed all the variables that were approaching significance to be selected and then systematically removed if a variable did not add to the significance of the model overall. Odds ratios (ORs) predicted the odds of having a higher NIHSS score in association with a combined thrombolytic and cholesterol reduction therapy.

In the regression model, the dependent variable, the NIHSS score stratification, assessed the degree of stroke severity. The primary independent variables in the regression model for the entire AIS population included all the demographic and clinical risk factors for the rtPA and prior cholesterol reducer use group compared to the rtPA without prior cholesterol reducer use group. The odds of developing worsening neurological functions (NIHSS score > 7) and for making progress or improvements (NIHSS score ≤ 7) were analyzed separately for the group who received rtPA (independent of cholesterol reducer use status), rtPA without cholesterol reducer use, and rtPA with cholesterol reducer use. Multicollinearity to determine interactions among independent variables was analyzed using the Hosmer-Lemeshow test. Overall correct classification percentage and area under the Receiver Operating Curve (ROC) determined the sensitivity, specificity, and accuracy of the logistic regression model. Logistic regression determined odd ratios and 95% confidence intervals (95% CI) of outcome measures. These odds ratios predicted which independent variables positively or negatively influenced a patient with an AIS to make progress or improvement in neurological functions.

## Results

A total of 5469 AIS patients were identified. In this population, 1608 patients were eligible for rtPA and 1327 of these received rtPA (Table 1). Of the patients treated with rtPA, 630 were taking a cholesterol reducer prior to the event, whereas 687 were not. As presented in Table 1, the rtPA treated subset of patients were younger (65.8 ± 14.8 vs. 67.7 ± 14.7), more likely to be Hispanic (2.3% vs. 1.3%) and have a higher BMI (28.18 ± 7.01 vs. 28.84 ± 6.84). This group presented with lower rates of carotid stenosis (4.2% vs. 6.7%) and diabetes (31.3% vs. 36.7%) but had higher rates of depression (15.4% vs. 12.5%) and dyslipidemia (52.8% vs. 49.6%). Furthermore, the rtPA group was more likely to be on HRT (2.3% vs. 1.2%), suffer from migraines (3.4% vs. 2.1%), and be obese (51.1 vs. 39.4). This group presented with fewer prior strokes (21.9% vs. 27.4%), but a greater history of TIAs (10.8 vs. 8.1%). They were less likely to have PVD (6.0% vs. 7.7%) and chronic renal disease (6.0% vs. 8.9%) but were more likely to be taking cholesterol reducing agents (47.6% vs. 43.4%) and antidepressants (16.7% vs. 11.8%). The rtPA group was taking fewer antidiabetic medications (24.9% vs. 28.1%) and presented with higher NIHSS scores (9.95 ± 6.6 vs. 7.82 ± 6.8). This group also presented with lower levels of serum cholesterol (168.7 ± 46.5 mg/dL vs. 173.0 ± 53.6 mg/dL), creatinine concentrations (1.14 ± 0.75 mg/dL vs. 1.34 ± 1.27 mg/dL), and blood glucose (141.3 ± 74.8 mg/dL vs. 149.2 ± 82.9 mg/dL) when compared to the non rtPA group. Moreover, the rtPA group differed significantly on ambulation classification prior to stroke, on admission, and on discharge. Patients in the rtPA group were more likely to be directly admitted to the hospital (23.4% vs. 19.9%) and have an improved ambulatory status (55.3% vs. 29.7%).

**Table 1:** Demographic and clinical characteristics of ischemic stroke patients receiving rtPA or not receiving rtPA. Results for continuous variables are presented as Mean ± SD, while discrete data are presented as percentage frequency. Pearson’s Chi-Square is used to compare differences between demographic and clinical characteristics in rtPA treated groups

Characteristic	No rtPA group	rtPA group	***P***-value
Number of patients	4142	1327	
Age Group: No. (%)			
< 50	467 (11.3)	191 (14.4)	0.001*^a^
50–59	736 (17.8)	260 (19.6)	
60–69	981 (23.7)	318 (24.0)	
70–79	942 (22.7)	289 (21.8)	
> =80	1016 (24.5)	269 (20.3)	
Mean ± SD	67.7 ± 14.7	65.8 ± 14.8	<.001*^b^
Race: No (%)			
White	3228 (77.9)	1060 (79.9)	0.313
Black	774 (18.7)	228 (17.2)	
Other	140 (3.4)	39 (2.9)	
Gender: No. (%)			
Female	1994 (48.1)	668 (50.3)	0.163
Male	2148 (51.9)	659 (49.7)	
Hispanic Ethnicity: No. (%)	55 (1.3)	30 (2.3)	0.017*^a^
BMI: Mean ± SD	28.18 ± 7.01	28.84 ± 6.84	0.003*^b^
Medical History: No. (%)			
Atrial Fib	713 (17.2)	211 (15.9)	0.266
Coronary Artery Disease	1262 (30.5)	399 (30.1)	0.782
Carotid Artery Stenosis	278 (6.7)	56 (4.2)	0.001*^a^
Depression	516 (12.5)	205 (15.4)	0.005*^a^
Diabetes	1520 (36.7)	415 (31.3)	< 0.001*^a^
Drugs or Alcohol	260 (6.3)	77 (5.8)	0.532
Dyslipidemia	2055 (49.6)	700 (52.8)	0.047*^a^
Stroke Family History	364 (8.8)	130 (9.8)	0.265
Heart Failure	453 (10.9)	137 (10.3)	0.531
Hormonal Replacement Therapy	48 (1.2)	31 (2.3)	0.002*^a^
Hypertension	3262 (78.8)	1044 (78.7)	0.95
Migraine	89 (2.1)	45 (3.4)	0.011*^a^
Obesity	1633 (39.4)	678 (51.1)	< 0.001*^a^
Previous Stroke	1134 (27.4)	290 (21.9)	< 0.001*^a^
Previous TIA (> 24 h)	334 (8.1)	143 (10.8)	0.002*^a^
Prosthetic Heart Valve	52 (1.3)	10 (0.8)	0.133
Peripheral Vascular Disease	321 (7.7)	79 (6.0)	0.029*^a^
Chronic Renal Disease	368 (8.9)	79 (6.0)	0.001*^a^
Sickle Cell	4 (0.1)	0 (0.0)	0.257
Sleep Apnea	125 (3.0)	45 (3.4)	0.495
Smoker	1098 (26.5)	388 (29.2)	0.052
Medication History: No (%)			
HTN medication	2851 (68.8)	943 (71.1)	0.125
Cholesterol Reducer	1796 (43.4)	632 (47.6)	0.006*^a^
Diabetes medication	1164 (28.1)	331 (24.9)	0.025*^a^
Antidepressant	489 (11.8)	222 (16.7)	< 0.001*^a^
Initial NIHSS Score: No (%)			
0–9	2556 (76.3)	733 (59.4)	< 0.001*^a^
10–14	308 (9.2)	199 (16.1)	
15–20	308 (9.2)	193 (15.6)	
21–25	180 (5.4)	110 (8.9)	
Mean ± SD	7.43 ± 8.11	10.55 ± 8.18	< 0.001*^b^
Lab values: Mean ± SD			
Total cholesterol	173.01 ± 53.6	168.66 ± 46.48	0.006*^b^
Triglycerides	139.25 ± 104.13	140.71 ± 107.94	0.674
HDL	41.77 ± 13.91	41.8 ± 13.65	0.946
LDL	105.39 ± 42.06	102.52 ± 39.07	0.029*^b^
Lipids	6.64 ± 2.83	6.25 ± 1.6	< 0.001*^b^
Blood Glucose	149.22 ± 82.86	141.29 ± 74.84	0.001*^b^
Serum Creatinine	1.34 ± 1.27	1.14 ± 0.75	< 0.001*^b^
Initial Platelet Count	36,527.8 ± 87,310.62	174,859.69 ± 128,632.87	< 0.001*^b^
INR	1.17 ± 0.57	1.06 ± 0.15	< 0.001*^b^
Vital Signs: Mean ± SD			
Heart Rate	82.07 ± 18.94	81.81 ± 17.18	0.644
Blood Pressure Systolic	151.99 ± 30.04	151.31 ± 26.92	0.439
Blood Pressure Diastolic	82.29 ± 19.38	82.92 ± 18.3	0.277
Ambulation Status Prior to Event: No. (%)			
Ambulate Independently	3628 (87.6)	1259 (94.9)	< 0.001*^a^
Ambulate with Assistance	180 (4.3)	23 (1.7)	
Unable to Ambulate	193 (4.7)	20 (1.5)	
Not Documented	140 (3.4)	25 (1.9)	
Ambulation Status on Admission: No. (%)			
Ambulate Independently	1099 (26.5)	232 (17.5)	< 0.001*^a^
Ambulate with Assistance	1320 (31.9)	306 (23.1)	
Unable to Ambulate	1249 (30.2)	479 (36.1)	
Not Documented	474 (11.4)	310 (23.4)	
Ambulation Status on Discharge: No. (%)			
Ambulate Independently	1516 (36.6)	658 (49.6)	< 0.001*^a^
Ambulate with Assistance	1466 (35.4)	354 (26.7)	
Unable to Ambulate	847 (20.4)	222 (16.7)	
Not Documented	19 (0.5)	4 (0.3)	
First Care Received: No. (%)			
Emergency Department	3285 (80.1)	1012 (76.6)	0.006*^a^
Direct Admission	815 (19.9)	309 (23.4)	
**Improved Ambulation: No. (%)**	1141 (29.7)	684 (55.3)	< 0.001*^a^
**NIHSS > 7: No. (%)**	1177 (33.5)	690 (52.4)	< 0.001*^a^

Notes

^a^Pearson’s Chi-Squared test.

^b^Student’s T test.

* *P*-value < 0.05.

Table 2 shows the clinical and demographic characteristics associated with improving or worsening neurologic functions for patients who received rtPA and previously used cholesterol reducers versus those who didn’t have a history of cholesterol reducer therapy. In the rtPA with prior cholesterol reducer use group, patients with worsening neurologic functions were older (70.83 ± 13.26 vs. 65.95 ± 12.09), less likely to be Caucasian (86.3% vs. 79.0%), and more likely to be female (51.5% vs. 40.5%), with higher rates of atrial fibrillation (23.9% vs. 11.5%) and heart failure (15.2% vs. 9.7%). This group presented with lower rates of a family history of stroke (7.1% vs. 14.0%) and obesity (47.2% vs. 58.3%). For laboratory values, the group with worsening neurologic functions who received rtPA presented with higher blood glucose levels (147.04 ± 73.66 vs. 136.22 ± 63.35), higher international normalized ratio (INR) (1.07 ± 0.12 vs. 1.04 ± 0.12) and were more likely to be directly admitted to the hospital (84.5% vs. 67.3%). For the rtPA without prior cholesterol reducer use group, the patients with worsening neurologic functions were older (66.31 ± 15.82 vs. 59.67 ± 15.47), presented with lower BMI (27.83 ± 6.24 vs. 29.07 ± 6.72), higher rates of heart failure (10.8% vs. 5.2%), hypertension (71.1% vs. 59.5%), and were more likely to be taking anti-hypertensive (anti-HTN) medications (56.7% vs. 49.0%). This group presented with lower rates of a family history of stroke (6.3% vs. 12.7%), migraines (2.1% vs. 6.2%), obesity (45.4% vs. 56.2%) smoking history (29.1% vs. 38.2%) and triglycerides (128.33 ± 95. vs. 153.54 ± 119.4), but higher serum creatinine (1.16 ± 1.04 vs. 1.04 ± 0.4) and higher INR (1.07 ± 0.18 vs. 1.04 ± 0.15). In addition, this group presented with a higher heart rate (84.98 ± 18.94 vs. 80.71 ± 16.21) and were more likely to be directly admitted to the hospital (82.2% vs. 72.2%).

**Table 2:** Demographic and clinical characteristics of ischemic stroke patients treated with rtPA in combination with a cholesterol reducer, or no cholesterol reducer and with worsening neurologic functions (NIHSS score > 7) and neurologic improvements (NIHSS score ≤ 7). Results for continuous variables are presented as Mean ± SD, while discrete data are presented as percentage frequency. Pearson’s Chi-Square is used to compare differences between demographic and clinical characteristics in groups with a NIHSS score > 7 and NIHSS score ≤ 7 who received rtPA and either took or did not take a cholesterol reducer

	rtPA with Cholesterol Reducer			rtPA without Cholesterol Reducer		
Characteristic	NIHSS ≤ 7	NIHSS > 7		NIHSS ≤ 7	NIHSS > 7	
Number of patients	**321**	**309**	**P-value**	**306**	**381**	***P*-Value**
Age Group: No. (%)						
< 50 years	32 (10.0)	20 (6.5)	< 0.001*^a^	79 (25.8)	60 (15.7)	< 0.001*^a^
50–59	59 (18.4)	44 (14.2)		75 (24.5)	80 (21.0)	
60–69	103 (32.1)	73 (23.6)		71 (23.2)	67 (17.6)	
70–79	81 (25.2)	76 (24.6)		49 (16.0)	80 (21.0)	
> =80	46 (14.3)	96 (31.1)		32 (10.5)	94 (24.7)	
Age Mean ± SD	65.95 ± 12.09	70.83 ± 13.26	< 0.001*^b^	59.67 ± 15.47	66.31 ± 15.82	< 0.001*^b^
Race: No (%)						
White	277 (86.3)	244 (79.0)	0.050*^a^	243 (79.4)	287 (75.3)	0.446
Black	37 (11.5)	56 (18.1)		54 (17.6)	80 (21.0)	
Other	7 (2.2)	9 (2.9)		9 (2.9)	14 (3.7)	
Gender: No. (%)						
Female	130 (40.5)	159 (51.5)	0.006*^a^	159 (52.0)	205 (53.8)	0.630
Male	191 (59.5)	150 (48.5)		147 (48.0)	176 (46.2)	
Hispanic Ethnicity: No. (%)	7 (2.2)	8 (2.6)	0.737	5 (1.6)	10 (2.6)	0.377
BMI: Mean ± SD	29.72 ± 6.67	28.94 ± 7.72	0.180	29.07 ± 6.72	27.83 ± 6.24	0.014*^b^
Medical History: No. (%)						
Atrial Fib	37 (11.5)	74 (23.9)	< 0.001*^a^	35 (11.4)	62 (16.3)	0.070
Coronary Artery Disease	135 (42.1)	135 (43.7)	0.679	50 (16.3)	74 (19.4)	0.296
Carotid Artery Stenosis	24 (7.5)	17 (5.5)	0.315	4 (1.3)	11 (2.9)	0.159
Depression	58 (18.1)	60 (19.4)	0.664	39 (12.7)	48 (12.6)	0.954
Diabetes	135 (42.1)	124 (40.1)	0.623	66 (21.6)	86 (22.6)	0.753
Drugs or Alcohol	14 (4.4)	19 (6.1)	0.314	15 (4.9)	28 (7.3)	0.188
Dyslipidemia	273 (85.0)	265 (85.8)	0.800	75 (24.5)	81 (21.3)	0.312
Stroke Family History	45 (14.0)	22 (7.1)	0.005*^a^	39 (12.7)	24 (6.3)	0.004*^a^
Heart Failure	31 (9.7)	47 (15.2)	0.034*^a^	16 (5.2)	41 (10.8)	0.009*^a^
Hormonal Replacement Therapy	10 (3.1)	7 (2.3)	0.510	8 (2.6)	6 (1.6)	0.338
Hypertension	291 (90.7)	289 (93.5)	0.182	182 (59.5)	273 (71.7)	0.001*^a^
Migraine	11 (3.4)	7 (2.3)	0.382	19 (6.2)	8 (2.1)	0.006*^a^
Obesity	187 (58.3)	146 (47.2)	0.006*^a^	172 (56.2)	173 (45.4)	0.005*^a^
Previous Stroke	90 (28.0)	90 (29.1)	0.762	46 (15.0)	62 (16.3)	0.657
Previous TIA (> 24 h)	45 (14.0)	41 (13.3)	0.784	25 (8.2)	30 (7.9)	0.887
Prosthetic Heart Valve	2 (0.6)	4 (1.3)	0.386	1 (0.3)	3 (0.8)	0.430
Peripheral Vascular Disease	25 (7.8)	25 (8.1)	0.888	12 (3.9)	16 (4.2)	0.855
Chronic Renal Disease	23 (7.2)	24 (7.8)	0.774	11 (3.6)	21 (5.5)	0.236
Sleep Apnea	16 (5.0)	15 (4.9)	0.940	9 (2.9)	5 (1.3)	0.133
Smoker	88 (27.4)	69 (22.3)	0.140	117 (38.2)	111 (29.1)	0.012*^a^
Medication History: No (%)						
HTN medication	286 (89.1)	283 (91.6)	0.291	150 (49.0)	216 (56.7)	0.045*^a^
Diabetes medication	117 (36.4)	106 (34.3)	0.574	45 (14.7)	60 (15.7)	0.706
Antidepressant	63 (19.6)	64 (20.7)	0.734	44 (14.4)	51 (13.4)	0.708
Lab values: Mean ± SD						
Total cholesterol	155.76 ± 41.36	153.88 ± 46.06	0.598	184.85 ± 46.97	178.75 ± 43.84	0.087
Triglycerides	149.43 ± 115.24	133.22 ± 100.57	0.067	153.54 ± 119.4	128.33 ± 95.18	0.003*^b^
HDL	39.51 ± 11.76	40.65 ± 12.61	0.250	42.49 ± 15.41	44.19 ± 14.03	0.140
LDL	91.22 ± 34.93	88.74 ± 37.96	0.404	116.22 ± 38.58	112.65 ± 37.26	0.230
Lipids	6.37 ± 1.61	6.33 ± 1.45	0.727	6.17 ± 1.69	6.17 ± 1.65	0.973
Blood Glucose	136.22 ± 63.35	147.04 ± 73.66	0.049*^b^	138.19 ± 81.28	143.76 ± 79.88	0.372
Serum Creatinine	1.15 ± 0.54	1.21 ± 0.79	0.233	1.04 ± 0.4	1.16 ± 1.04	0.036*^b^
Initial Platelet Count	159,087.99 ± 131,106.09	170,621.77 ± 120,785.15	0.519	196,175.97 ± 133,607.09	175,315.17 ± 128,742.52	0.245
INR	1.04 ± 0.12	1.07 ± 0.12	0.005*^b^	1.04 ± 0.15	1.07 ± 0.18	0.016*^b^
Vital Signs: Mean ± SD						
Heart Rate	79.62 ± 15.03	81.31 ± 17.38	0.191	80.71 ± 16.21	84.98 ± 18.94	0.002*^b^
Blood Pressure Systolic	150.34 ± 25.3	150.94 ± 26.19	0.768	151.62 ± 28.49	152.87 ± 27.51	0.561
Blood Pressure Diastolic	81.42 ± 16.2	80.44 ± 17.35	0.465	84.92 ± 19.27	84.82 ± 19.41	0.941
Ambulation Status Prior to Event: No. (%)						
Ambulate Independently	315 (98.1)	276 (89.3)	< 0.001*^a^	297 (97.1)	361 (94.8)	0.038*^a^
Ambulate with Assistance	2 (0.6)	9 (2.9)		7 (2.3)	5 (1.3)	
Unable to Ambulate	2 (0.6)	10 (3.2)		1 (0.3)	7 (1.8)	
Not Documented	2 (0.6)	14 (4.5)		1 (0.3)	8 (2.1)	
Ambulation Status on Admission: No. (%)						
Ambulate Independently	93 (29.0)	17 (5.5)	< 0.001*^a^	95 (31.0)	24 (6.3)	< 0.001*^a^
Ambulate with Assistance	98 (30.5)	50 (16.2)		104 (34.0)	52 (13.6)	
Unable to Ambulate	18 (5.6)	198 (64.1)		21 (6.9)	238 (62.5)	
Not Documented	112 (34.9)	44 (14.2)		86 (28.1)	67 (17.6)	
Ambulation Status on Discharge: No. (%)						
Ambulate Independently	224 (69.8)	86 (27.8)	< 0.001*^a^	217 (70.9)	128 (33.6)	< 0.001*^a^
Ambulate with Assistance	76 (23.7)	98 (31.7)		78 (25.5)	100 (26.2)	
Unable to Ambulate	12 (3.7)	88 (28.5)		9 (2.9)	111 (29.1)	
Not Documented	1 (0.3)	1 (0.3)		0 (0.0)	2 (0.5)	
First Care Received: No. (%)						
Emergency Department	216 (67.3)	261 (84.5)	< 0.001*^a^	221 (72.7)	310 (82.2)	0.003*^a^
Direct Admission	105 (32.7)	48 (15.5)		83 (27.3)	67 (17.8)	
Improved Ambulation: No (%)	177 (56.5)	151 (55.3)	0.763	162 (53.3)	193 (56.5)	0.399

Notes

^a^Pearson’s Chi-Squared test.

^b^Student’s T test.

* *P*-value < 0.05.

**Fig. 1:**
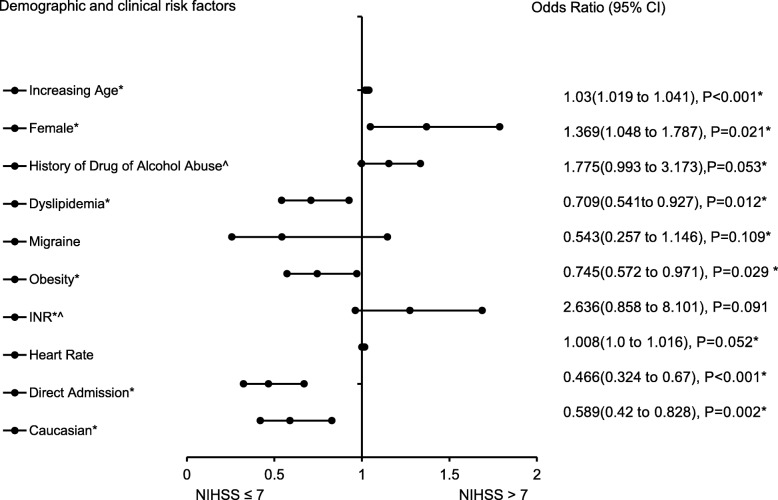
Forest Plot representation of clinical factors that were associated with the severity of stroke for the total ischemic stroke population with rtPA, independent of whether they received a cholesterol reducer. Adjusted OR < 1 denote factors that are associated with neurologic improvements (NIHSS score ≤ 7) while OR > 1 denote factors that are associated with worsening neurologic functions (NIHSS score > 7). Hosmer-Lemeshow test (*P* = 0.203), Cox & Snell (*R*^*2*^ = 0.100). The overall classified percentage of 61.8% was applied to check for fitness of the logistic regression model. *Indicates statistical significance (*P* < 0.05) with a 95% confidence interval. ^Indicates that data were modified by taking the 5th square root

**Fig. 2:**
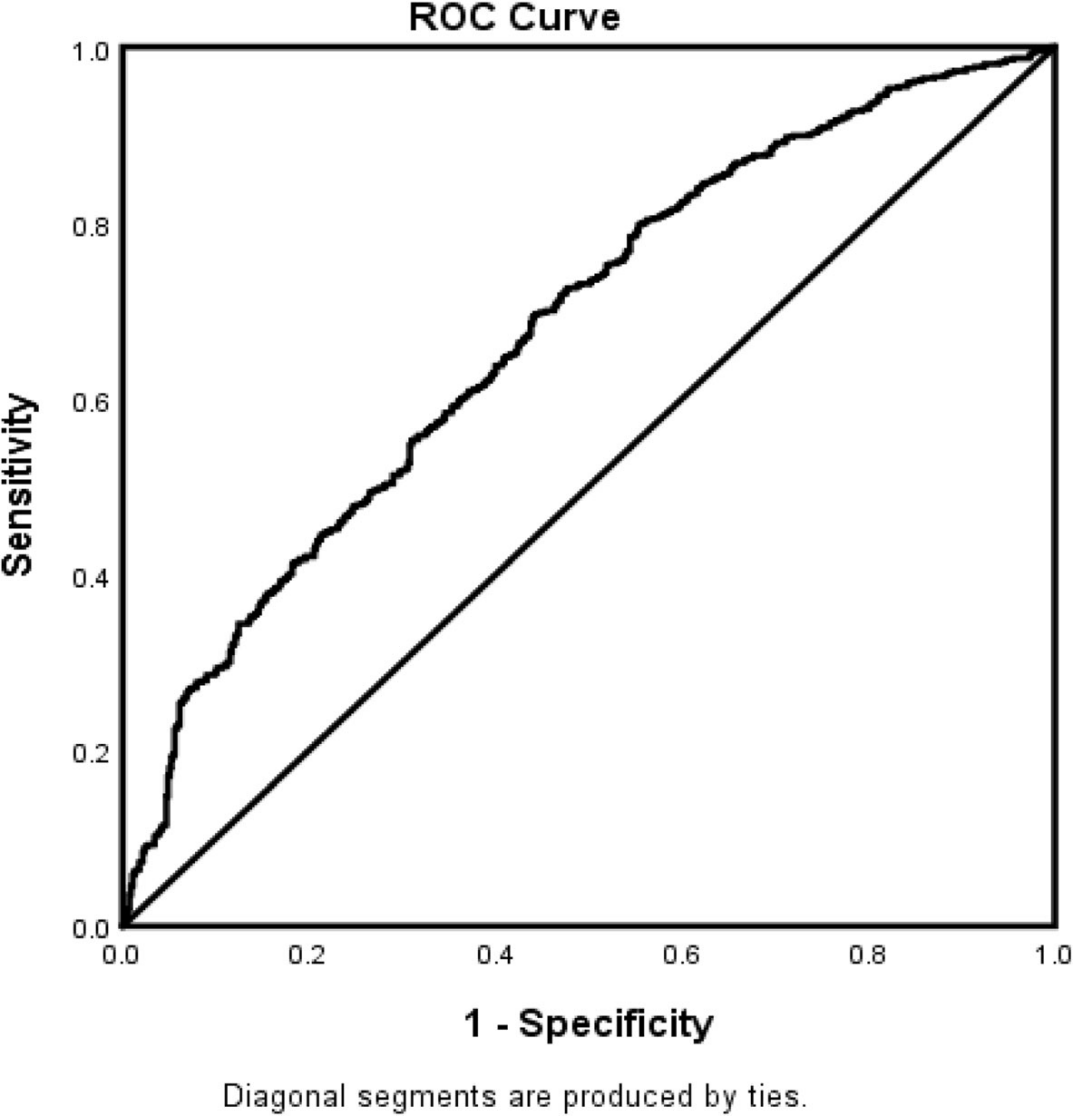
The ROC curve for the predictive power of the regression model for AIS population with rtPA. Higher area under the curve (AUC) values in ROC analysis indicate better discrimination of the score for the measured outcome. Classification table (overall correctly classified percentage = 61.8%) and area under the ROC curve (AUC = 0.677, 0.646–0.707) were applied to check model fitness. ^Indicates that data were modified by taking the 5th square root

**Fig. 3:**
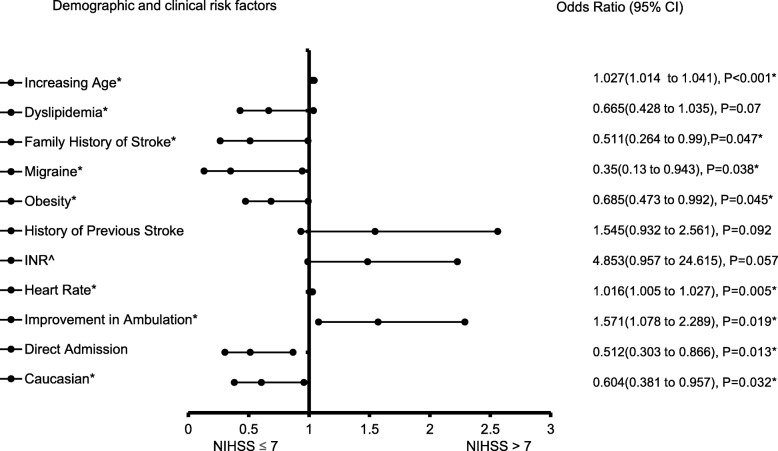
Forest Plot representation for clinical factors that were associated with stroke severities in ischemic stroke population with rtPA only. Adjusted OR < 1 denote factors that are associated with neurologic improvements (NIHSS score ≤ 7) while OR > 1 denote factors that are with worsening neurologic functions (NIHSS score > 7). Hosmer-Lemeshow test (*P* = 0.907), Cox & Snell (*R*^*2*^ = 0.123). The overall classified percentage of 65.7% was applied to check for fitness of the logistic regression model. *Indicates statistical significance (*P* < 0.05) with a 95% confidence interval. ^Indicates that data were modified by taking the 5th square root

**Fig. 4:**
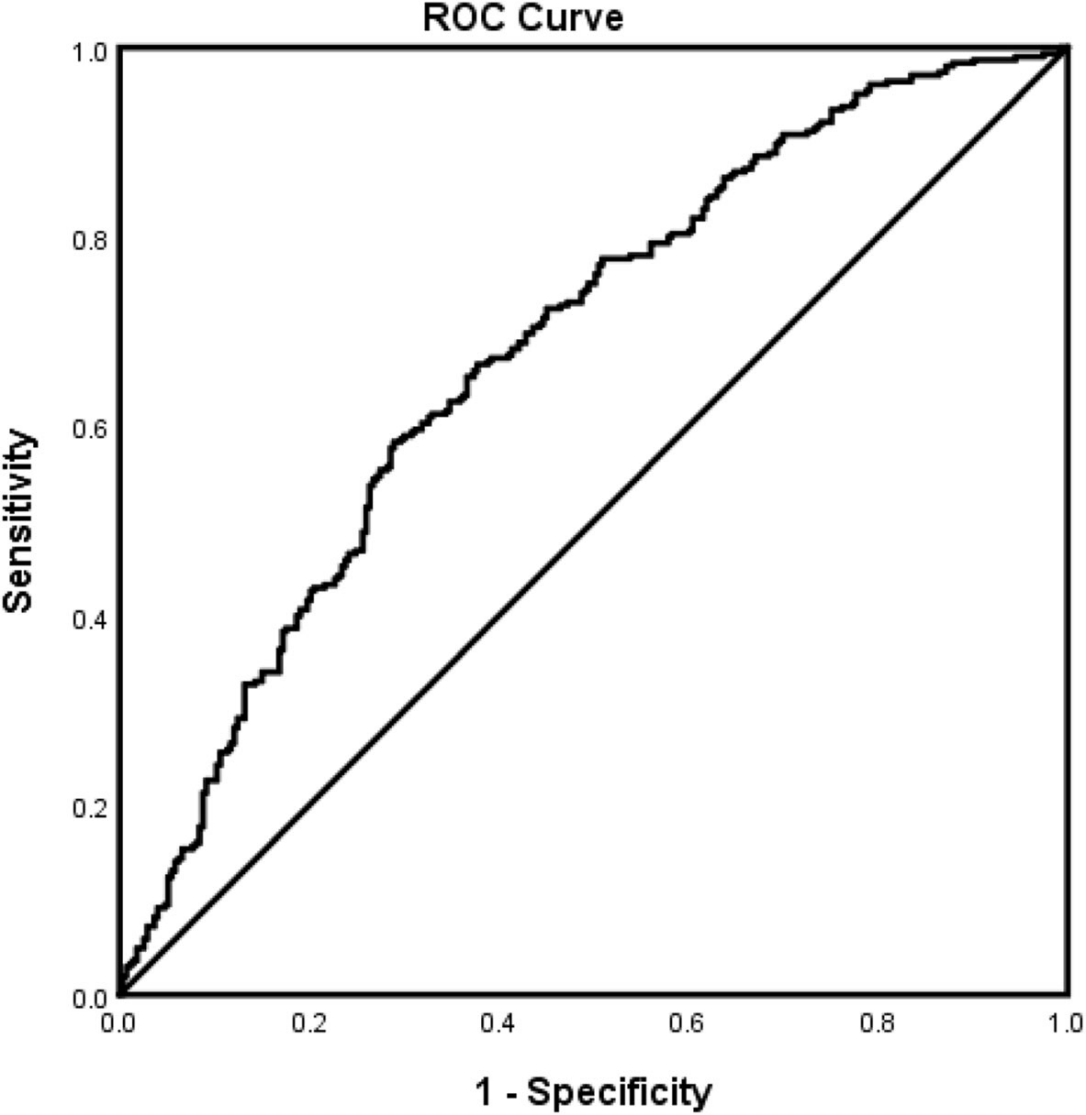
The ROC curve for the predictive power of the regression model AIS population who received rtPA and were not taking a cholesterol reducer. Higher area under the curve (AUC) values in ROC analysis indicate better discrimination of the score for the measured outcome. Classification table (overall correctly classified percentage = 65.7%) and area under the ROC curve (AUC = 0.681, 0.637–0.724) were applied to check model fitness. ^Indicates that data were modified by taking the 5th square root

**Fig. 5:**
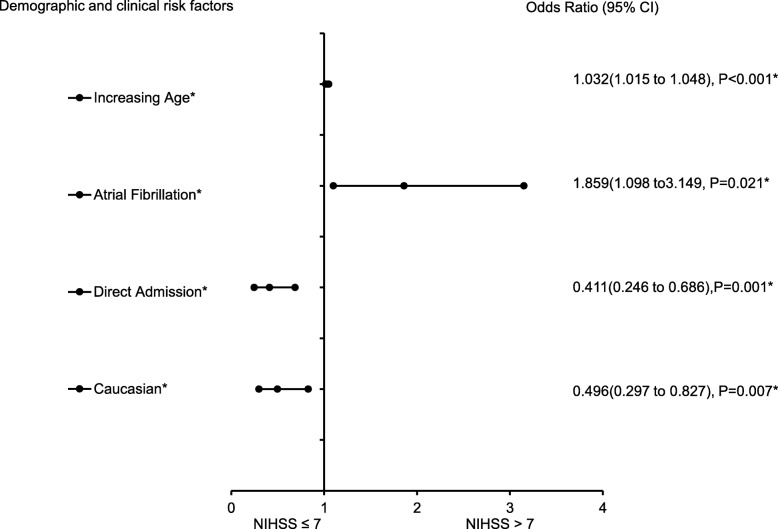
Forest Plot representation of clinical factors that were associated with stroke severities for ischemic stroke population with rtPA and taking a cholesterol reducer. Adjusted OR < 1 denote factors that are associated with neurologic improvements (NIHSS score ≤ 7) while OR > 1 denote factors that are with worsening neurologic functions (NIHSS score > 7). Hosmer-Lemeshow test (*P* = 0.415), Cox & Snell *(R*^*2*^ = 0.097). The overall classified percentage of 64.9% was applied to check for fitness of the logistic regression model. *Indicates statistical significance (*P* < 0.05) with a 95% confidence interval. ^Indicates that data were modified by taking the 5th square root

**Fig. 6:**
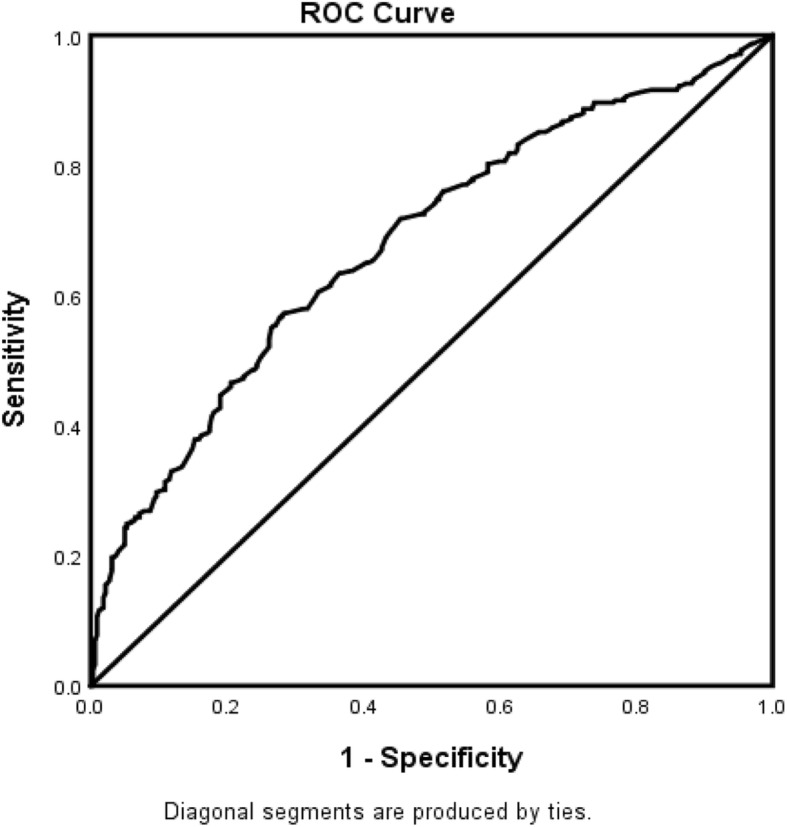
The ROC curve for the predictive power of the regression model for AIS population who received rtPA and were taking a cholesterol reducer. Higher area under the curve (AUC) values in ROC analysis indicate better discrimination of the score for the measured outcome. Classification table (overall correctly classified percentage = 64.9%) and area under the ROC curve (AUC = 0.680, 0.639–0.722) were applied to check model fitness. ^Indicates that data were modified by taking the 5th square root

The forest plot demonstrating clinical factors that were associated with the severity of stroke for the total ischemic stroke population (Fig. 1). In the adjusted analysis, increasing age (OR = 1.03, 95% CI, 1.019–10.4, *P* < 0.001) and being female (OR = 1.369, 95% CI, 1.048–1.787, *P* = 0.021) were variables associated with worsening neurologic functions, while dyslipidemia (OR = 0.709, 95% CI, 0.541–0.927, *P* = 0.012), obesity (OR = 0.745, 95% CI, 0.572–0.971, *P* = 0.029), direct admission (OR = 0.466, 95% CI, 0.324–0.670, *P* < 0.001), and Caucasian race (OR = 0.589, 95% CI, 0.42–0.828, *P* = 0.002) were associated with improving neurologic functions. The discriminating capability of the model was moderately strong as shown by the ROC curve (Fig. 2), with area under the curve (AUROC) = 0.677 (95% CI, 0.646–0.707, *P* < 0.001). In the rtPA group without prior cholesterol reducer use (Fig. 3) increasing age (OR = 1.027, 95% CI, 1.014–1.041, *P* < 0.0.01), higher heart rate (OR = 1.016, 95% CI, 1.005–1.027, *P* = 0.005), and improvement in ambulation (OR = 1.571, 95% CI, 1.078–2.289, *P* = 0.019) were associated with neurologic deterioration or worsening neurologic functions. Patients with a family history of stroke (OR = 0.511, 95% CI, 0.264–0.99, *P* = 0.047), migraines (OR = 0.250, 95% CI, 0.130–0.943, *P* = 0.038), obesity (OR = 0.685, 95% CI, 0.473–0.992, *P* = 0.045), direct admission (OR = 0.512, 95% CI, 0.303–0.866, *P* = 0.013), and Caucasian race (OR = 0.604, 95% CI, 0.381–0.957, *P* = 0.032) were more likely to be associated with a neurologic improvement. As presented in Fig. 4, the predictive power of the logistic regression was moderately strong, as the area under the curve (AUROC) was 0.681 (95% CI, 0.637–0.724, *P* < 0.001). For the rtPA group with prior cholesterol reducer use (Fig. 5), increasing age (OR = 1.032, 95% CI, 1.015–1.048, *P* < 0.001) and atrial fibrillation (OR = 1.859, 95% CI, 1.098–3.149, *P* = 0.021) were more likely to be associated with worsening neurologic function. Direct admission (OR = 0.411, 95% CI, 0.246–0.686, *P* = 0.001) and Caucasian race (OR = 0.496, 95% CI, 0.297–0.827, *P* = 0.007) were associated with greater neurologic improvement. The ROC curve for the predictive power of the regression model as presented in Fig. 6 shows that the discriminating capability of the model was moderately strong, with the area under the curve (AUROC) = 0.680 (95% CI, 0.639–0.722, *P* < 0.001).

## Discussion

Cholesterol reducers, particularly statins, are known to protect against ischemic injury and could be beneficial in the acute phase of stroke [28], but it remains controversial whether a combined statin and thrombolytic therapy increases the risk of hemorrhage, a major rtPA-related complication. Moreover, specific baseline clinical risk factors may interact with the combined statin and thrombolytic therapy to worsen neurologic functions in AIS patients. The current study investigated specific factors that may contribute to favorable or unfavorable outcomes in a combined statin and thrombolytic therapy.

Our finding indicates that AIS patients without prior cholesterol reduction therapy but received rtPA were more likely to be elderly patients with higher heart rate and are more likely to improve neurologically fowling rtPA therapy. Moreover, AIS patients, with a previous history of stroke, migraines, obesity, direct admission, and Caucasian ethnicity were more likely to exhibit neurologic improvement following thrombolytic therapy. These findings are consistent with a single-center study involving small cohorts which shows that a history of stroke [47, 48], migraines [49], obesity [50, 51] and direct admission into the neurological unit [52] were associated with improved functional outcomes following thrombolytic therapy. In addition, our finding that AIS patients without prior cholesterol reducer use who presented with increased age and higher heart rate had a likelihood of worsening neurological function following thrombolytic shows consistency with previous studies [9, 53, 54].

In the combined rtPA with prior cholesterol reducer use cohort, the effect of increasing age and atrial fibrillation was significant for a worsening neurological function in the AIS patients. Interestingly, studies have shown that rtPA works as effectively in the elderly as in younger patients [55–57]. It has also been shown that age may influence the outcome and risk of hemorrhage after rtPA therapy [58–60]. Therefore, increasing age is associated with an increased number of comorbidities and worse outcomes regardless of rtPA-related complications [61–63]. While studies have not shown age alone to negate the beneficial effects of rtPA, the benefits of statins treatment in the elderly is controversial because neurological outcome is associated with many clinical and demographic factors. For example, elderly presents with more comorbidities usually receive a complex treatment regimen consisting of different classes of drugs which may interact with statins and rtPA resulting in worsening neurological outcomes. Therefore, the mechanisms associated with poor outcomes in the interaction between cholesterol reducers and rtPA-treated in elderly AIS patients’ needs further evaluation in order to develop management strategies to prevent poor outcomes in elderly AIS patients taking a combined rtPA and statin therapy.

The observed worsening neurologic function in AIS patients with atrial fibrillation treated with the combined cholesterol reducer and rtPA therapy is not surprising as atrial fibrillation has been shown to be associated with worse initial outcomes in patients with ischemic strokes [41, 64]. Statins are known to be effective at reducing the incidence of atrial fibrillation after coronary surgery, but the effectiveness of statins on management of atrial fibrillation is not clear [65]. It has been shown that the development of atrial fibrillation can be due to a NO-redox imbalance in the atria [66]. However, there are two different mechanisms that have been reported to determine whether atrial fibrillation is acute or chronic [66]. Statins in this study [66] were shown to be effective in providing protection from the development of acute atrial fibrillation, but once chronic atrial fibrillation is established, statins were not effective in the long-term management.

Treatment with statins before or early after an ischemic stroke is reported to trigger favorable outcomes [9] through its pleiotropic effects, such as endothelial nitric oxide increase [67], reduction of oxidative stress [68], inhibition of platelet activation [69], and anti-inflammatory activities [70]. These properties suggest statins as a safe and promising pre- or cotreatment with rtPA for an effective reperfusion therapy in the acute phase of stroke [7, 71], However, these benefits are not attributed to a statin’s cholesterol lowering effects but are associated with its other pleiotropic effects. Observational studies on cholesterol reducers including statins [72] indicate an inverse correlation of cholesterol and intracerebral hemorrhage (ICH) risk. This finding indicates that statin treatment maybe associated with an increased risk of ICH after stroke when combined with rtPA [73]. Statins inhibit the generation of thrombin and disrupts fibrinolytic balance by upregulating endogenous rtPA production and reducing plasminogen activator-inhibitor 1 expression [74]. This results in an enhanced efficacy of rtPA which increases the risk of ICH and other vascular events. Overall, this combination can lead to the induction of several events including ischemic, hemorrhagic activities as well as impairment of vascular events linked with atrial fibrillation which will significantly decrease the chances of good outcome AIS [75]. While cholesterol reducers, and more specifically statins, may be ineffective in the long-term management of atrial fibrillation, combining statin and rtPA treatment should not be denied to patients with AIS patients with atrial fibrillation. Rather, a decision to combine statin and rtPA for AIS patients with atrial fibrillation can be done after clinical stabilization with appropriate risks and benefits.

One of the main strengths of the study is the ability to use a logistic regression model to identify baseline demographic and clinical risk factors that may determine whether AIS patients with prior cholesterol reduction therapy and rtPA therapy with baseline stroke severity are more likely to exhibit neurological improvement or develop worsening neurologic function. Our findings indicate that prior cholesterol reducer usage, namely a statin, in addition to rtPA therapy may be associated with worsening neurologic function for elderly AIS patients with atrial fibrillation, while the statin and rtPA combination in Caucasians and those directly admitted to a neurology unit are more likely to be associated with improvement in neurologic function. This study also presents some limitations. Because of the retrospective nature of this study, bias should always be considered when trying to draw conclusions. Moreover, while the stroke registry did record the data for patients that take statins versus those who did not, information about other cholesterol reducers and on any patients that suffer health declines of any kind after stopping statin therapy was not included in the data analysis. Therefore, it is difficult to compare the effects that may be due to another type of cholesterol reducer apart from statins or due to the potential heterogenous pool of cholesterol reduction therapy used. Finally, since the stroke registry was only obtained from one hospital, it is challenging to make conclusions about the entire population. However, this study has shown specifically that prior statin use in combination with rtPA therapy in patients with AIS is a promising therapy for Caucasians at early admission only, necessitating more clinical trials in the near future.

## Conclusion

After controlling for demographic and clinical risk factors, older patients with atrial fibrillation who presented with an AIS on pre-stroke cholesterol reduction therapy, namely a statin, and received rtPA were more likely to develop worsening neurologic functions, while Caucasian patients directly admitted to a neurology unit were more likely to develop improving neurologic functions.

## Supplementary information

**Additional file 1.** A conceptual diagram of our findings implications and underlying mechanism

## Data Availability

The retrospective datasets are available by request from the corresponding author of this manuscript.

## Abbreviations

Adjusted OR: Adjusted odd ratio
BMI: Body mass index
CHF: Congestive heart failure
CI: Confidence interval
INR: International normalized ratio
LDL-C: Low-density lipoprotein
rtPA: Recombinant tissue plasminogen
ICH: intracerebral hemorrhage
TC: Total cholesterol
TG: Total glyceride
AIS: Acute ischemic stroke
NIHSS: National institute of health stroke scale
MRI: Magnetic resonance imaging
CT: Computer tomography
MCA: Middle cerebral artery
CAD: Coronary artery disease
HRT: Hormone replacement therapy
TIA: Trans ischemic attack
PVD: Peripheral vascular disease
ROC: Receiver operating curve
INR: International normalized ratio
