# Supplementary material for: Cholesterol reducer and thrombolytic therapy in acute ischemic stroke patients
Source: Lipids Health Dis. 2020 May 6;19:84. doi: 10.1186/s12944-020-01270-2 (PMC7201805; doi:10.1186/s12944-020-01270-2)
Supplement: Supplementary file 1 — Additional file 1. A conceptual diagram of our findings implications and underlying mechanism [file 12944_2020_1270_MOESM1_ESM.pptx]

## Slide 1
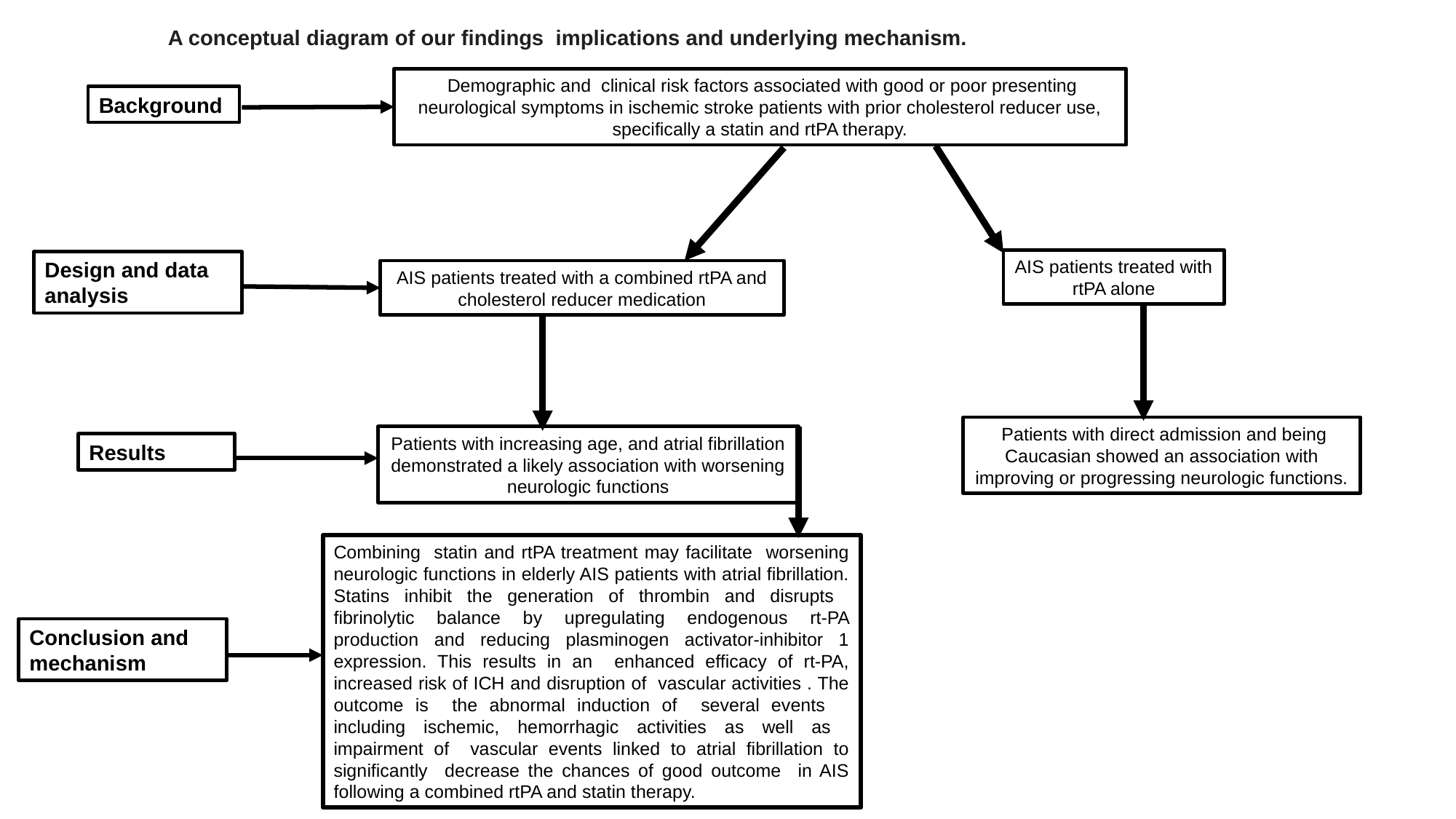

A conceptual diagram of our findings implications and underlying mechanism.
 Demographic and clinical risk factors associated with good or poor presenting neurological symptoms in ischemic stroke patients with prior cholesterol reducer use, specifically a statin and rtPA therapy.
Background
AIS patients treated with rtPA alone
Design and data analysis
AIS patients treated with a combined rtPA and cholesterol reducer medication
 Patients with direct admission and being Caucasian showed an association with improving or progressing neurologic functions.
Patients with increasing age, and atrial fibrillation demonstrated a likely association with worsening neurologic functions
Results
Combining statin and rtPA treatment may facilitate worsening neurologic functions in elderly AIS patients with atrial fibrillation. Statins inhibit the generation of thrombin and disrupts fibrinolytic balance by upregulating endogenous rt-PA production and reducing plasminogen activator-inhibitor 1 expression. This results in an enhanced efficacy of rt-PA, increased risk of ICH and disruption of vascular activities . The outcome is the abnormal induction of several events including ischemic, hemorrhagic activities as well as impairment of vascular events linked to atrial fibrillation to significantly decrease the chances of good outcome in AIS following a combined rtPA and statin therapy.
Conclusion and mechanism
